# Structural Insights into LDPE/UHMWPE Blends Processed by γ-Irradiation

**DOI:** 10.3390/polym15030696

**Published:** 2023-01-30

**Authors:** Traian Zaharescu, Nicoleta Nicula, Maria Râpă, Mihai Iordoc, Violeta Tsakiris, Virgil Emanuel Marinescu

**Affiliations:** 1 INCDIE ICPE CA, 3131 Splaiul Unirii, 030138 Bucharest, Romania; 2 Faculty of Materials Science and Engineering, University Politehnica of Bucharest, 313 Splaiul Independentei, 060042 Bucharest, Romania

**Keywords:** UHMWPE, LDPE, compatibility, radiation effects, structural modifications

## Abstract

Ultra-high-molecular-weight polyethylene (UHMWPE) matrices containing low-density polyethylene (LDPE), hydroxyapatite (HAp) as filler, and rosemary extract (RM) as stabilizer were investigated for their qualification for long-term applications. The significant contributions of the blend components were analyzed, and variations in mechanical properties, oxidation strength, thermal behavior, crystallinity, and wettability were discussed. SEM images of microstructural peculiarities completed the introspective survey. The stability improvement due to the presence of both additives was an increase in the total degradation period of 67% in comparison with an unmodified HDPE/UHMWPE blend when the materials were subjected to a 50 kGy γ-dose. There was growth in activation energies from 121 kJ mol^−1^ to 139 kJ mol^−1^ when HAp and rosemary extract delayed oxidation. The exposure of samples to the action of γ-rays was found to be a proper procedure for accomplishing accelerated oxidative degradation. The presence of rosemary extract and HAp powder significantly increased the thermal and oxidation resistances. The calculation of material lifetimes at various temperatures provided meaningful information on the wearability and integrity of the inspected composites.

## 1. Introduction

The manufacture of reinforced polymer materials has become common practice in the manufacture of improved composites with foreseen durability [1,2,3]. UHMWPE is a versatile material whose various purposes include biomedical achievements [4,5], drug delivery [6], manufacture of orthopedical devices [7], and wearable products [8] and are sustained by satisfactory mechanical properties and chemical resistance, good friction coefficients, availability for compounding, and, in essence, good durability. For the extension of long-term durability and improvement in material toughness, the peroxide treatment of UHMWPE promotes the crosslinking of macromolecules in the presence of vitamin E [9]. However, a promising alternative for the efficient sterilization and the promotion of surface modification of UHMWPE matrices is radiation processing [10,11]. Recent investigations on radiation-exposed UHMWPE [12,13] have emphasized that this kind of processing initiates the formation of several alkyl radicals further converted into oxygen-centered radicals after their reactions with diffused oxygen. The understanding of the long-term stability of γ-irradiated UHMWPE is based on the generated radicals and their decay after a maximum of 10 h from the elapsing of irradiation [14]. The dependence of consumption rates of polyenyl radicals on the crystallinity degree of UHMWPE is correlated with the diffusion rate of oxygen, which feeds oxidative degradation [15]. Accordingly, the behaviors of various sorts of ultra-high-molecular-weight polyethylene matrices follow various kinetics because the unlikely spreading of radicals between the crystalline and amorphous phases influences the local abundance of degradation products formed from the inhomogeneous distribution of former radicals. An analysis of the fragmentation process occurring during the high-energy exposure of UHMWPE revealed that each dose of 15 kGy created 2 mmol of macroradicals, and the extraction of a proton by one peroxyl radical required an activation energy of 108 kJ mol^−1^ [16]. In fact, the degradation mechanism described previously states that the main oxidation products (alcohols, ketones, acids, peracids) appeared by the reactions of hydroperoxides, whose concentrations grew proportionally with applied dose [16].

Hydroxyapatite-containing UHMWPE composites were explored for the identification of the interaction degrees among components and the evaluation of inorganic phase contribution to the improved stability of the host matrix [17]. Radiation-induced processes in polymer matrices have not been explored fully.

The association of various sorts of polyethylene with UHMWPE can determine a reinforcement of blend compositions that can be easily compatibilized with several inorganic fillers, such as hydroxyapatite [18,19], carbon nanotubes [20], graphene [21], inorganic oxides [22], nanoclays [23], and many others. All these hybrid systems confirm the availability of the basic component for intimate, compatibilized materials destined for a large variety of applications requiring high durability. Oxidative resistance can be greatly increased by the addition of an antioxidant. Some detailed studies have been achieved with vitamin E, which depict the provided good protection against aging during operation or over shelf life [24,25].

The availability of polyethylene to generate high-performance blends has been comprehensively studied [26,27]. Accordingly, some procedures through which the compatibilization of blending components makes possible the achievement of intimate phase interpenetration during radiation processing have been published [28,29,30]. The structure and morphology of various sorts of polyethylene are the favorable features by which polyethylene is modified due to the action of radiolysis-free radicals and their contribution to forming intermolecular bridges [31]. The challenge position of γ-treatment on polyethylene blends is motivated by the high number of technologies by which new polymer materials are converted into materials with expected properties [32].

The association of two types of polyethylene, LDPE and UHMWPE, is based on the scission of these molecules [33,34], the formation of free radicals, and their recombination to form new, crosslinked material [35]. Improvement in oxidation resistance has previously been studied by the addition of biological stimulators [25,36,37], which efficiently protect polyolefin against aging in biological environments. The medical applications of this tandem polyethylene are guaranteed by the presence of appropriate antioxidant structures [38]. Of course, high-energy irradiation is a convenient technological procedure through which molecular scission is promoted without difficulties, and it does not need any additional reagent for the assistance of crosslinking.

The utilization of natural antioxidants for the oxidation protection of irradiated polymers, especially material destined for medical purposes, is a suitable solution because they are biologically compatible with the human body, their content of active component is enough high, and their price is quite accessible [39]. According to our praxis on a range of antioxidants, rosemary extract is a proficient additive that efficiently blocks the oxidation of polymers by means of polyphenolic composition [40,41]. The conversion of rosmarinic acid into various related structures, such as rosmanol or carnosol [41,42], enables long-term protection and convenient thermal stability in aggressive environments, such as biological surroundings.

In this paper, the effects of component loadings, the presence of rosemary in sample formulations, and the irradiation conditions are presented. The discussion is turned to the contribution of γ-processing to the evolution of material states, where the complementary evaluation methods provide information for the application of radiation technologies. This study investigates the extension of γ-exposure to sterilization applications, to the fabrication of medical wear such as scaffolds, and to the conversion of polyethylene waste into useful products by high-energy methods. The investigated systems and investigation approach may be considered as new intakes by which the compatibilization technique, the association of the two additives, and the applied evaluation background open a new direction for the large-scale manufacture of ecological materials.

## 2. Materials and Methods

### 2.1. Materials

**Figure 1:**
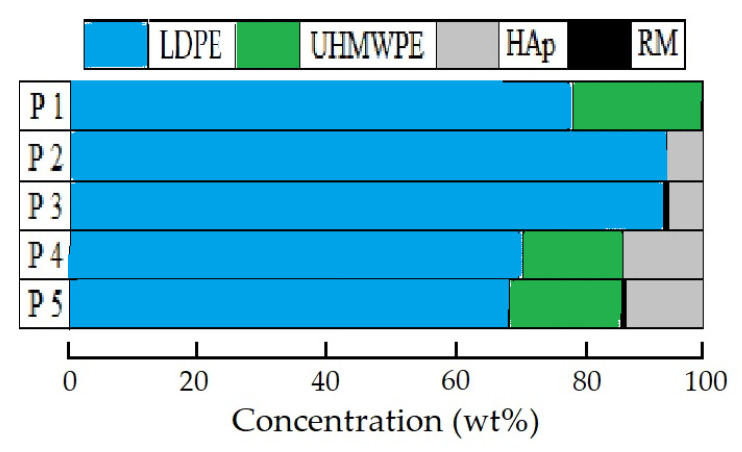
Illustration of component loadings for studied patterns.

All the studied material compositions are described in detail in a previous paper of this series [27]. A picture illustrating the blending ratios of the components, including low-density polyethylene (LDPE), ultra-high-molecular-weight polyethylene (UHMWPE), hydroxyapatite (HAp), and rosemary extract (RM), is shown in Figure 1.

### 2.2. Preparation of Samples

The details on the preparation of the investigated samples were reported earlier [27]. All preparatory conditions, including name, size, and weight, were previously specified. The results obtained for the unirradiated probes were analyzed in the first part of this series [27]. However, sample preparation by solvent (CHCl_3_) evaporation at room temperature, the addition of HAp and rosemary powders after homogenization of the polymer solutions, and the gentle drying of liquid aliquots in aluminum caps were the main stages accomplished until the specimens were irradiated and measured. The final weights of dry samples were placed in the range of 3–5 mg.

### 2.3. Irradiation

Exposure to the action of γ-radiation was accomplished in an irradiation machine (Ob Servo Sanguis, Budapest, Hungary) by applying the continuous rotation of a hosting can for the homogenous exposure of γ-processed samples. The applied dose rate was 0.5 kGy h^−1^. The environment of radiation processing was achieved at room temperature. Four irradiation doses were selected for the characterization of component interactions, namely 0, 25, 50, and 100 kGy. Greater values are usually applied in the technological processing of polymers, while smaller ones (25 and 50 kGy) may be considered extremely valuable techniques for the radiation sterilization of plastics when an irradiated material allows it without degradation. All the measurements were performed immediately after the elapse of irradiation, avoiding the modification of radical distributions over time.

### 2.4. Chemiluminescence

The evaluation of the thermal and radiation stabilities was carried out with a LUMIPOL 3 chemiluminescence spectrometer (Institute of Polymers, Slovak Academy of Sciences, Bratislava, Slovakia) according with the description of procedures [43]. Two types of measurements were applied: nonisothermal determinations were conducted at four heating rates (3.7, 5.0, 10.0, and 15.0 °C min^−1^) for characterizing thermal behavior under various diffusion rates, while isothermal determinations were performed at 170 °C. This temperature was a convenient value where the degradation maintained a reasonable rate to be accurately evaluated. The aluminum caps where the dry investigating materials were placed did not interfere during photon emission because they were inert relative to the thermal quanta emission.

**Figure 2:**
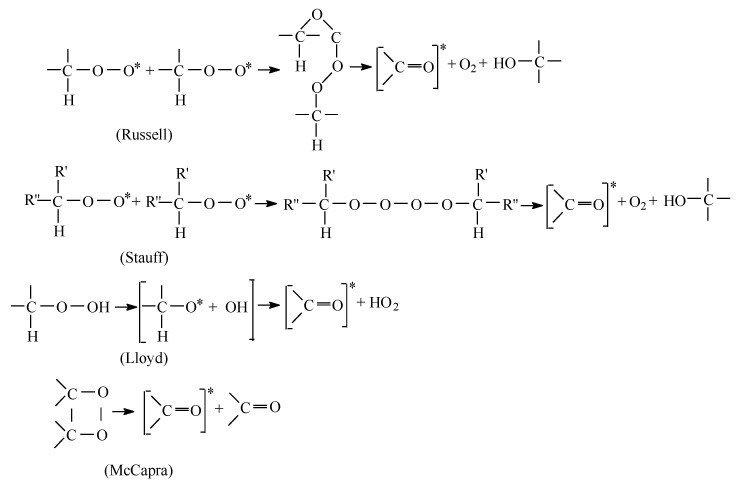
The mechanisms of chemiluminescence emission.

The mechanisms of chemiluminescence emission are presented in Figure 2, where different considerations on the interpretation of emission sources lead to similar comments on the development of degradation.

For the characterization of material stability, two kinetic parameters, namely t_1/2_ (the moment when the rate of radical generation equals the rate of decay) and t_max_ (the maximum oxidation time) were taken into consideration.

### 2.5. FTIR Spectroscopy

The FTIR spectral investigation was achieved with a JASCO 4200A (JASCO, Tokyo, Japan) spectrometer. The spectra were recorded in the region of carbonyl vibration (1720 cm^−1^), which described the evolution of oxidation in correlation with the chemiluminescence measurements. The spectral records were the result of 48 scans with a satisfactory resolution of 4 cm^−1^.

### 2.6. DSC

The thermal analysis of the present composites was performed by means of a DSC 823e calorimeter (Mettler Toledo, Switzerland) at a suitable heating rate of 10 °C min^−1^. The crystallinity degree was calculated with Equation (1):

(1) $$X_{c}=\frac{\Delta H_{m}}{\Delta H_{100\%,polyolefin}}\times 100\%$$

where Δ*H_m_* is the measured enthalpy of the melted blends (J g^−1^), and Δ*H*_100%, *polyolefin*_ is the enthalpy of melting for 100% crystalline LDPE (289.74 J g^−1^) [44].

### 2.7. Mechanical Testing

The mechanical survey was achieved by testing conducted on the polymer samples using an LFM 30 kN Walter & Bai AG Switzerland testing machine at room temperature according to BS EN ISO 6892-1:2009. Tensile testing was realized on rectangular samples with dimensions of Lxlxh = 40 mm × 10 mm × 2 mm at a speed of 5 mm/min, performing 5 tests for each sample. The average values are presented as the final results.

### 2.8. Contact Angle

The contact angle measurements were conducted with a lab-made device composed of an xyz mobile table and a DinoLite microscope at ambient temperature (22 ± 1 °C) and relative humidity (20−40%) [2]. A separate testing syringe was dedicated for each test liquid to avoid crosscontamination. A liquid droplet of about 3 μL was formed at the end of each syringe and carefully deposited onto the sample surface. The contact angle was calculated with DinoLite software. The reported contact angle values represented the average of 5 measurements from both left and right sights. DI water produced by means of a Millipore system was used in this study. Ethylene glycol (99.8%) and glycerol (99%) were used as received from Sigma-Aldrich.

The contact angle measurement of a pure liquid presenting known surface tension and surface tension parameters with respect to a given solid surface is the common way to evaluate the surface energy that characterizes the interactions of solid material. The surface free energy (σ*_S_*) of a solid is defined as the change in the total surface free energy (*G*) per surface area (*A*) at constant temperature (*T*), pressure (*P*), and moles (*n*) [45]:

(2) $$\sigma_{S}={\left(\delta G/\delta A\right)}_{T,P,n}$$

The Owens–Wendt theory (also sometimes referred to as the “harmonic mean” method) was developed to account for specific (polar-type) interactions between solid surfaces and liquids. Owens and Wendt envisioned the surface energy of a solid as being comprised of two parts: a dispersive component and a polar component. The dispersive component theoretically accounts for van der Waals and other non-site-specific interactions that a surface is capable of establishing with applied liquids. On the other hand, the polar component theoretically accounts for dipole–dipole, dipole-induced dipole–hydrogen bonding, and other site-specific interactions that a surface can establish with applied liquids. Based on this idea, Owens and Wendt developed a two-parameter model for describing surface interactions in contrast with the one-parameter model reported by Zisman [46].

Mathematically, the theory is based on two fundamental equations, which describe the contact interactions between solid surfaces and liquids. The equations are as follows:

- Good’s equation

(3) $$\sigma_{SL}=\sigma_{S}+\sigma_{L}-2{\left(\sigma_{L}^{D}\sigma_{S}^{D}\right)}^{1/2}-2{\left(\sigma_{L}^{p}\sigma_{S}^{P}\right)}^{1/2}$$

- Young’s equation

(4) $$\sigma_{S}=\sigma_{SL}+\sigma_{L}\cos\theta$$

where *σ_L_* is the overall surface tension of the wetting liquid, *σ_L_^D^* is the dispersive component of the surface tension for the wetting liquid, *σ_L_^P^* is the polar component of the wetting liquid, *σ_s_* is the overall surface energy of the studied solid, *σ_s_^D^* is the dispersive component of the surface tension for the solid, *σ_s_^P^* is the polar component of the surface tension energy of the studied solid, *σ_SL_* is the interfacial tension between the solid and liquid phases, and *θ* is the contact angle between the two contacted materials.

The Owens–Wendt relationship combines the equations of Good and Young to generate Equation (5):

(5) $$\frac{\sigma_{L}(\cos\theta +1)}{2{\left(\sigma_{L}^{D}\right)}^{1/2}}={\left(\sigma_{S}^{P}\right)}^{1/2}\frac{{\left(\sigma_{L}^{P}\right)}^{1/2}}{{\left(\sigma_{L}^{D}\right)}^{1/2}}+{\left(\sigma_{S}^{D}\right)}^{1/2}$$

Since the Owens–Wendt theory is a two-component model for solid surface energy, it is also a two-component model for liquid surface tension. The overall surface tension of each probe liquid must be separated into polar and dispersive components as well. This is achieved using a standard reference surface. The accepted standard reference surface for two-component liquid surface tension determination is poly(tetrafluoroethylene) (PTFE). Pure, untreated PTFE has a surface energy of 18.0 mJ m^−2^, assuming that it can establish no polar-type interactions. On the other hand, *σ_s_* = *σ_s_^D^* = 18.0 mJ m^−2^, as well as *σ_s_^P^* = 0 mJ m^−2^ for PTFE. By substituting these values into the primary Owens–Wendt equation, the dispersive surface tension (*σ_L_^D^*) can be determined for any liquid when the overall surface tension (*σ_L_*) is initially known. The real method of evaluation is the measurement of the contact angle between an investigated liquid and PTFE (*θ*_PTFE_). The polar surface energy component for a liquid is accordingly determined by the following difference: (*σ_L_^P^* = *σ_L_* − *σ_L_^D^*).

### 2.9. Biodegradability Testing

The assessment of polymeric composite biodegradability was performed by exposure to a fungi suspension (a mixture of *Aspergillus niger*, *Penicillium funiculosum*, *Paecilomyces variotii*, and *Chaetomium globosum* si *Gliocladium virens*) in the presence of Czapek-Dox nutritive medium in Petri dishes according to the ISO 846/2019 standard [47]. The incubation was conducted in conditions favorable to fungi germination and growth at T = 30 °C and RH = 90%. Fungal growth was observed at regular intervals of 7, 14, 21, and 28 days.

### 2.10. SEM

The SEM images were obtained with an FESEM–Auriga scanning electron microscope (Carl Zeiss, Germany). The selected experimental parameters were a 5 kV acceleration voltage with a working distance of 4.2–4.3 mm in a high-vacuum room. This tension value was appropriately required by the compensation accomplished with a nitrogen CC system that prevented the formation of an electronic cloud. Polymer samples embedded in epoxy resin with conductive properties were further polished and treated with permanganic (chemical solution) etching. The prepared surfaces were analyzed in an SESI (secondary electron secondary ions) detector chamber of an Everhart–Thornley-type model.

## 3. Results and Discussion

The high-energy irradiation of polymers inevitably produces molecular scissions where the looser bonds are places. Accordingly, the formation of free radicals initiates two main antagonist processes: crosslinking and degradation [48]. The availability of polyethylene to form homogenous and stable blends has seldom been confirmed [49,50]. As always demonstrated, polymers and polymer blends satisfy the requirements of society [51]. Radiation processing demonstrates constantly that exposure to high-energy radiation (accelerated electrons and γ-rays) provides pertinent solutions for the foreseen modifications [52]. In addition, the effects of high-energy treatments are always associated with improvement in oxidation resistance and, consequently, material durability by the crosslinking of available radical intermediates.

The γ-irradiation of blends consisting of LDPE and UHMWPE is a proper route to produce improved materials, which may have the required suitability for different ranges of application, including medical wear, scaffolds, pipes, cases, sheets, bottles, automotive items, and many others. Their thermal strength is obtained not only due to appropriate chemical structures, convenient morphology, functional properties, but also to the long-term resistance offered by stabilizers [53]. The homogeneity of γ-exposed materials is assured by spreading radicals in polymer matrices and high concentrations of radicals along the radiation track.

### 3.1. Chemiluminescence

**Figure 3:**
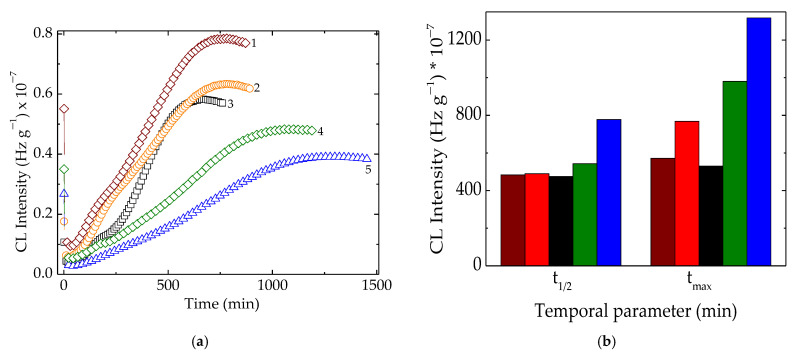
(**a**) Isothermal CL curves recorded on samples with different polyolefin compositions. (**b**) Temporal kinetic parameters of t_1/2_ and t_max_ characterizing the progress of oxidation in the studied samples. Testing temperature: 160 °C; irradiation dose: 50 kGy. Compositions: (1) LDPE/UHMWPE; (2) LDPE/HAp; (3) LDPE/HAp/RM; (4) LDPE/UHMWPE/HAp; (5) LDPE/UHMWPE/HAp/RM. The symbol colors are the same for both graphs. The colors of the bars have the same meanings as in (**a**).

Chemiluminescence evaluation of polymer stability is considered accurate insight into structural changes during degradation or molecular modifications [54]. After the γ-radiolysis of polymer matrices, the progress of oxidative degradation advances differently because the components have their own contribution to the fates of free radicals. In Figure 3, emerging oxidation was conducted in relation to the present fillers, which acted efficiently in the diminution of oxidation rates.

The kinetic analysis of the oxidation of these blends after being subjected to the action of γ-radiation revealed the consistent contributions of hydroxyapatite and rosemary powder, even when added separately or as an antioxidant couple. These two compounds showed individual stabilizing features offering the possibility to delay oxidation, but the material stability with the combined effect became much more relevant. The protection mechanisms are totally different. If the active components of rosemary extract, especially the majoritarian constituent of carnosic acid [55], act as the scavengers of free radicals by the substitution of phenolic-proton-like hindered phenols [56], hydroxyapatite delays oxidation by the adsorption and superficial blocking of the chain initiator in degradation development [2]. The evolution of oxidation described by the temporal parameters (Figure 3b) highlights the favorable contributions of the used fillers on the last stage of oxidation. In an earlier step of degradation when the generation rate for peroxyl radicals exceeded the rate of their consumption, the stabilization effect presented lower levels. If the oxidation process overtook the t_1/2_ moment (when the formation and decay rates were equal), the protection activities became more visible. It allowed for considering that the stabilization efficiency is more effective and the oxidation state of this kind of polymer material is maintained at a lower possible level. The simultaneous presence of both polyolefins, LDPE, and UHMWPE in the radiation-processed material indicated a good concern in the fabrication of high-performance products with a very long life time. The high values of the maximum oxidation times (980 min and 1318 min at 160 °C and 50 kGy for LDPE/UHMWPE/HAp and LDPE/UHMWPE/HAp/RM, respectively) proved the noteworthy intervention of the used fillers with propitious consequences on material durability. The solution of the found stability improvement demonstrates the present proposition as an ecological version of composition.

**Figure 4:**
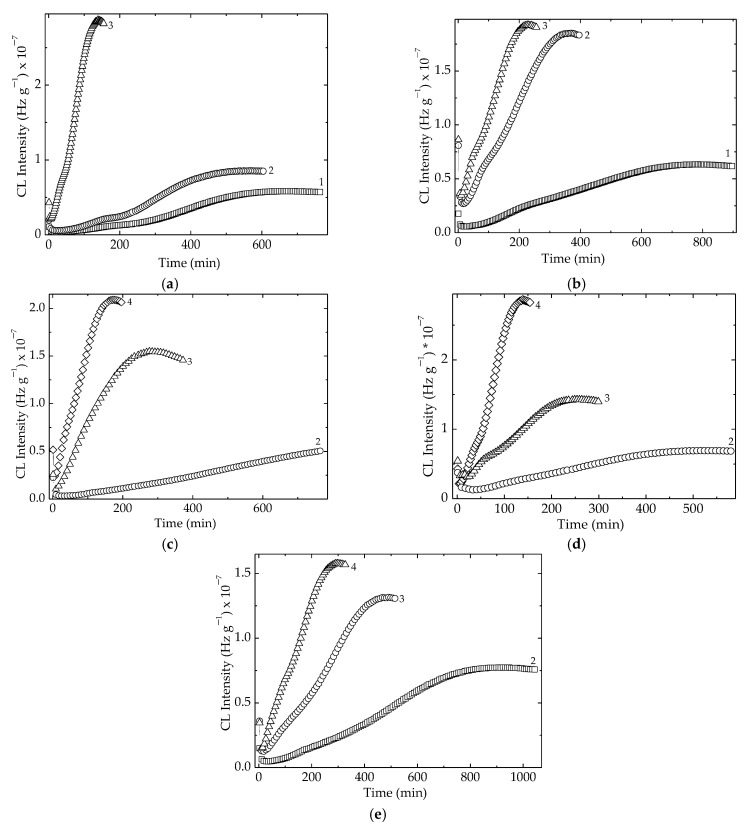
Isothermal CL spectra recorded for all the investigated samples. Temperatures: (1) 160 °C, (2) 170 °C, (3) 180 °C, (4) 190 °C. Irradiation dose: 50 kGy. (**a**) LDPE/UHMWPE; (**b**) LDPE/HAp; (**c**) LDPE/HAp/RM; (**d**) LDPE/UHMWPE/HAp; (**e**) LDPE/UHMWPE/HAp/RM.

As can be noticed from Figure 4, there were two temperature ranges that characterized the material stability. The first group, consisting of LDPE/UHMWPE and LDPE/Hap, needed smaller values of temperatures because their stability was somewhat lower. The second group, composed of LDPE/HAp/RM, LDPE/UHMWPE/Hap, and LDPE/UHMWPE/HAp/RM, needed elevated temperatures because their thermal resistances were more improved. The main reason for this behavior was not only the modification of crystallinity degree [57], but also the radiation crosslinking of the polymeric components or the presence of the stabilizing couple. It should be noted that the extension of oxidation induction time for the second group of compositions was also an indication of their stability over a larger temperature range.

The progress of oxidative degradation ran in concordance with the characteristic values of activation energies (Table 1). Since the investigated materials were subjected to γ-irradiation, they required less energy compared with pristine samples [27]. However, the values found were high enough to describe good thermal stability after radiation processing.

**Figure 5:**
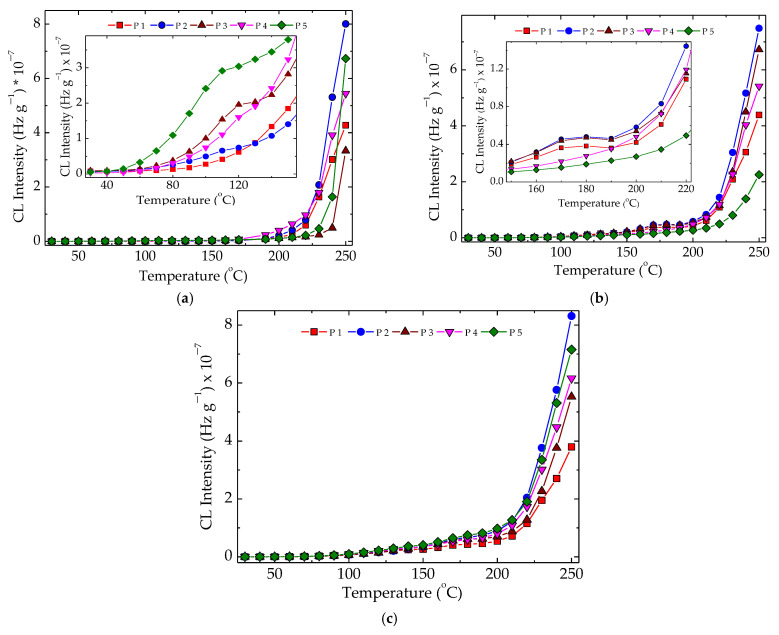
Nonisothermal CL spectra recorded for various compositions of LDPE/UHMWPE probes subjected to the action of γ-radiation. (P1) LDPE/UHMWPE; (P2) LDPE/HAp; (P3) LDPE/HAp/RM; (P4) LDPE/UHMWPE/HAp; (P5) LDPE/UHMWPE/HAp/RM. Exposure doses: (**a**) 0 kGy; (**b**) 50 kGy; (**c**) 100 kGy. Heating rate: 10 °C min^−1^.

The nonisothermal CL measurements, which provided the peculiar values of temperature for the start of oxidation, allowed visualizing the intimate structural modifications produced by the increase in temperature. These spectra presented in Figure 5 reveal some peculiarities related to the formation of peroxyl radicals as the initiators of oxidation in the mechanism of polymer aging [58]. The pristine samples, which were unirradiated specimens, presented a smooth evolution of degradation, where the hydroperoxides were instantly converted into oxygenated products and no accumulation of these initiators occurred (Figure 5a). At 50 kGy, when a radiation pretreatment led to the fragmentation of polymer chains, a shoulder appeared at 175 °C. It indicated the availability of hydroperoxides to initiate several degradation chains, continuing the degradation with a higher amplitude after this temperature was overtaken. The direct consequence of this behavior led to in increase in the values of onset oxidation temperature (Figure 5b). Longer exposure of the samples by irradiating them at 100 kGy decreased the stability of the materials, and the OIT values become smaller (Figure 5c). While the presence of hydroxyapatite tended to allow easier penetration of oxygen, which fed oxidation, the added rosemary extract acted as an efficient antioxidant. These contradictory directions placed the nonisothermal curves in certain orders that differed as the value of irradiation dose was changed.

**Figure 6:**
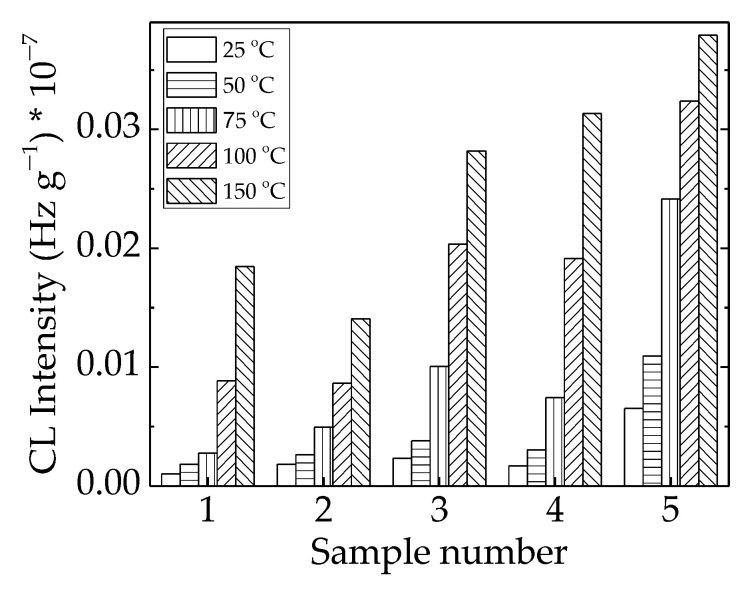
Histogram of CL intensities measured for LDPE/UHMWPE probes irradiated at 25 kGy. (1) LDPE/UHMWPE; (2) LDPE/HAp; (3) LDPE/HAp/RM; (4) LDPE/UHMWPE/HAp; (5) LDPE/UHMWPE/HAp/RM.

The implications of γ-irradiation in the modification of the studied polymer blends found possible applications in the recycling of polyethylene waste, as the final properties could be further improved, and the amelioration obtained was illustrated in the relevant functionality over a long period of time. The lack of structural changes at a low irradiation dose of 25 kGy (Figure 6) suggested that radiation sterilization is a proper treatment for implementation in microbiological cleaning.

These blends may serve as an example of quality improvement by means of high-energy technology assisted by nanofillers and natural antioxidants.

The chemiluminescence investigation proved that the compatibilization of LDPE and UHMWPE in stable blends may be achieved by pertinent γ-irradiation doses when appropriate compounds delay oxidation.

### 3.2. FTIR Spectroscopy

**Figure 7:**
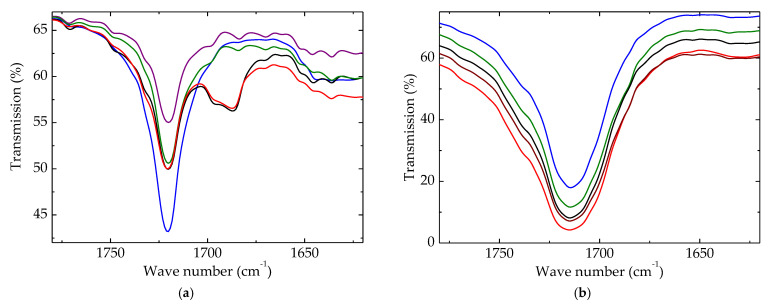
Carbonyl regions of FTIR spectra for polyethylene blends. Irradiation dose: (**a**) 0 kGy; (**b**) 100 kGy. (**--brown**) LDPE/UHMWPE; (**--red**) LDPE/HAp; (**--black**) LDPE/HAp/RM; (**--green**) LDPE/UHMWPE/HAp; (**--blue**) LDPE/UHMWPE/HAp/RM.

Advances in polymer degradation can be worthily described by the accumulation of degradation products containing carbonyl units. The initial peak was narrow, illustrating the presence of this function in the pristine material (Figure 7a). It was assumed that this peak may be explained by the presence of extract components or some impurities of HAp.

Upon the γ-radiolysis of polymeric components, the formation of carbonyl-containing structures is inevitable. The growth in the measured transmission values followed the accumulation of these compounds according with the existing compositional restrictions. Figure 7b proves that the coupling of HAp/RM was very efficient, and the amount of carbonyls reached the lowest level, even though the spectroscopic determination established a contrary situation.

During the operation period, these studied blends could be aged under energetic conditions, but the additives protected them, and their lives were accordingly extended. The important aspect to be considered is the preservation of the oxidation state when the coupling of HAp and RM effectively delayed the deterioration of the material condition.

### 3.3. DSC

**Figure 8:**
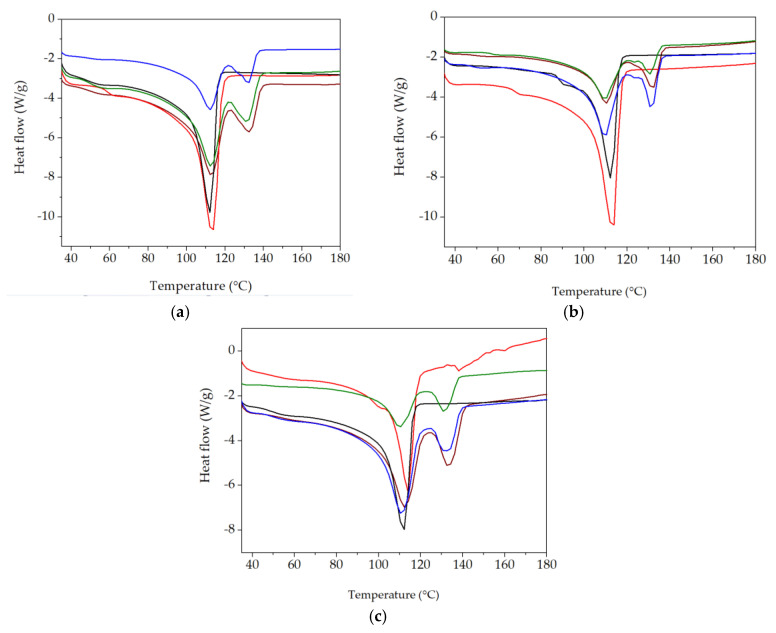
DSC thermograms for various LDPE-based composites (first heating scan) at 0 kGy (**a**), 50 kGy (**b**), and 100 kGy (**c**) (exo down). Sample compositions: brown) LDPE/UHMWPE; (**--red**) LDPE/Hap; (**--black**) LDPE/Hap/RM; (**--green**) LDPE/UHMWPE/Hap; (**--blue**) LDPE/UHMWPE/Hap/RM.

The application of this thermal analysis procedure is based on structural modifications, which are tracked by material response to heating processes [59,60]. The characterization of component behaviors during the compatibilization of the LDPE-UHMWPE mixtures was suggested by the evolution of the DSC curves in which the thermal features were envisaged. DSC curves for the LDPE-based composites investigated at different doses are shown in Figure 8. These DSC curves show the temperatures at which components were turned from solid to liquid states. The Tm of UHMPE was higher than the similar value for LDPE, which reflects individual thermal behaviors of the components. Because of the lamellar structure of UHMWPE [61], its penetration by LDPE was difficult. While the irradiation of the LDPE/UHMWPE composite with a γ-dose of 50 kGy led to a decrease in the first endothermic melting peak by about 2 °C compared to the unirradiated sample, the Tm of UHMWPE increased from 132.7 °C (initial sample) to 133.6 °C at a dose of 100 kGy. This composite exhibited a higher Xc at all doses (32.9% for 0 kGy, 38.4% for 50 kGy, and 38.8% for 100 kGy) compared to the other corresponding formulations due to the easier crystallization of lower-molecular-weight chains occurring as an effect of radiation-induced cleavage.

The simultaneous presence of both types of polyethylene was marked by separate peaks and revealed a clear separation of the phases and a weak interaction between them [62]. The thermal effects of the additives (hydroxyapatite and rosemary extract) are profoundly noticeable because the particles affected the interface energy [63]. These non-overlapping curves suggest different degrees of miscibility [64] when the added particles were surrounded by unlikely polymer entanglements.

**Figure 9:**
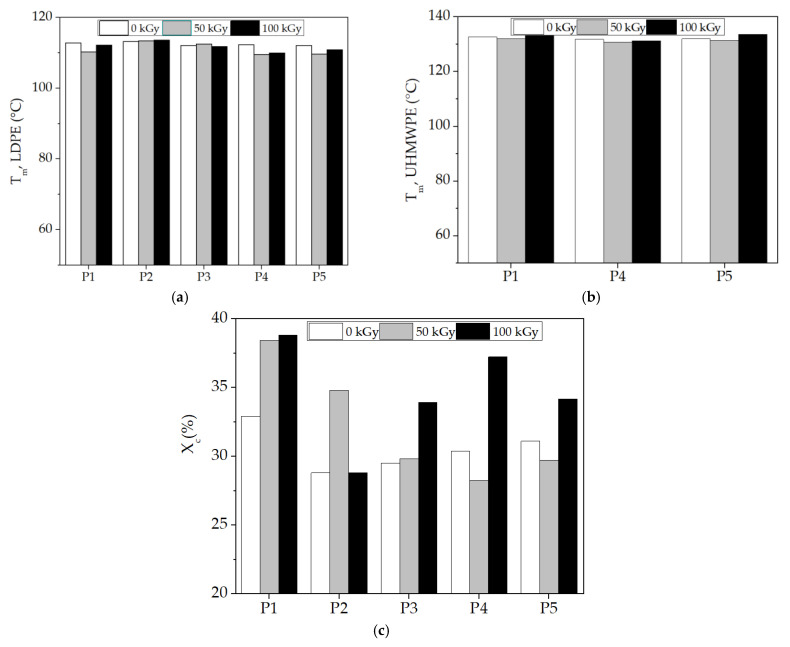
DSC parameters plotted as melting temperature at different γ-doses. (**a**) T_m_ for LPDE, (**b**) T_m_ for UHMWPE, and (**c**) total degree of crystallinity (X_c_). (P1) LDPE/UHMWPE; (P2) LDPE/Hap; (P3) LDPE/Hap/RM; (P4) LDPE/UHMWPE/Hap; (P5) LDPE/UHMWPE/Hap/RM.

The polyethylene blends studied by DSC presented some peculiarities that demonstrate certain compatibilization degrees due to the structural similarities in the molecular configurations. However, the different morphological aspects of components led to modifications in the compatibilization features (Figure 9). The effects of inorganic materials, such as Hap [65], as well as polyphenol types of natural antioxidants [66], cause different thermal responses related to interface cohabitation, where crystalline islands are spread through amorphous material. The presence of Hap in the composites led to a decrease in Xc values compared with the LDPE/UHMWPE composites at all the irradiation doses. For the LDPE/Hap composite, Xc increased by ~20% only at 50 kGy compared to the untreated sample due to the nucleation role of Hap [67]. At the dose of 50 kGy, decreases in both Xc and Tm associated with LDPE and UHMWPE were recorded as being accompanied in the LDPE/UHMWPE/Hap and LDPE/UHMWPE/Hap/RM composites. At this dose, the mobility of the polymer chain was higher, favoring the occurrence of radical decay. This behavior can be explained by “frozen free radicals” that hindered the chain mobility of radicals formed in the crystalline regions of the polyethylene during irradiation [68]. Similar effects of decreasing degree of crystallization with irradiation dose have been observed for blends of polyethylene/rosemary extract [69], poly(lactic acid) (PLA)/Hap [2], and UHMWPE/Vitamin E [70].

The introduction of RM into the composites led to a slow decrease in the Tm of LDPE due to a plasticizing effect. At the 100 kG dose (Figure 9), a decrease in the Tm of LDPE was observed for the LDPE/Hap/RM, LDPE/UHMWPE/Hap, and LDPE/UHMWPE/Hap/RM composites by about 2 °C compared to untreated composites. It was found that the Tm values of UHMWPE became higher for the LDPE/UHMWPE (133.6 °C) and LDPE/UHMWPE/Hap/RM (133.6 °C) composites at 100 kGy in comparison with the initial composite values (132.7 °C and 132 °C, respectively). It may be noticed that there was an evident increase in the Xc values for the LDPE/UHMWPE, LDPE/Hap/RM, LDPE/UHMWPE/RM, and LDPE/UHMWPE/Hap/RM composites at 100 kGy in comparison with the crystallinity degrees of the initial composites by 18%, 15%, 22%, and 10%, respectively. The presence of rosemary extract in the polyolefin matrix had a favorable effect on the inhibition of the reaction of free radicals with oxygen during radiation processing. It was accompanied by growth in the crystalline phase [71]. It was expected that the radical decay rate in the crystalline region would be very low due to the restriction in the macromolecular chains [72]. Higher crystallinity was associated with shorter chains that crystallized easier, leading to the radiation protection imparted by Hap and RM moieties. These finding were consistent with the chemiluminescence results (Figure 3).

As the results indicate, the thermal effects of the studied additives, Hap and RM, were important on the lifetimes of the materials, and their concentrations must be carefully selected so that the properties of final products must be equilibrated products with long-term applications.

### 3.4. Mechanical Testing

**Figure 10:**
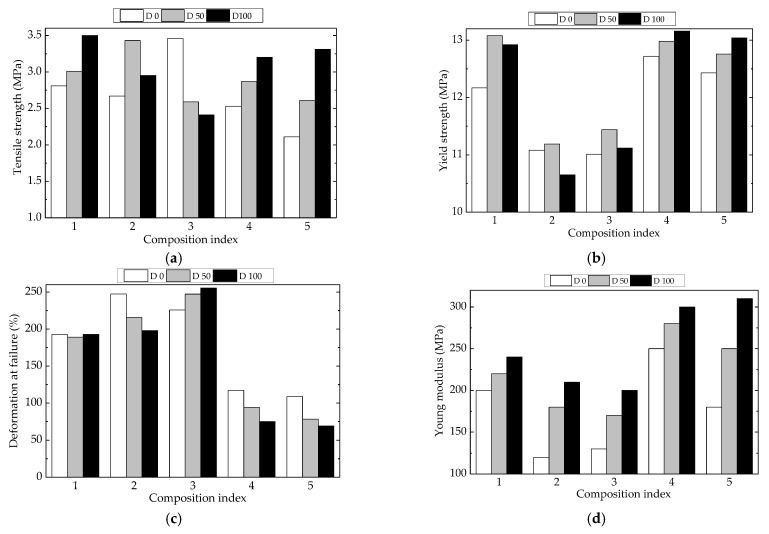
Mechanical properties for various LDPE-based composites at three processing doses. Types of samples: (1) LDPE/UHMWPE, (2) LDPE/HAp, (3) LDPE/HAp/RM, (4) LDPE/UHMWPE/HAp, (5) LDPE/UHMWPE/HAp/RM. (**a**) tensile strength, (**b**) yield stength, (**c**) deformation at failure, (**d**) Young modulus.

The characterization of material viability involves the description of mechanical properties that define the intimate cohesion and integrity of material. The availability of polyethylene to be crosslinked under the action of γ-radiation may be compared with the behavior of polyethylene structures containing a certain degree of unsaturation [73]. Figure 10 illustrates some mechanical properties measured for LDPE-based composites. The presence of Hap induced a slight modification in the continuity of the material, while rosemary extract was involved in the crosslinking tendencies due to free radical activities.

As it is known, the mechanical properties of composite materials are dependent on the type and quantity of filler materials in the basic matrix, their geometry, and their orientation, as well as the method of manufacturing the composite materials. The degree of interaction at the interface between components is one of the most important factors that affect the final performance of composite materials, with a strong degree of interaction having a beneficial effect on mechanical properties. From Figure 10, the tensile strength and yield strength showed the highest values for P1, P4, and P5 where UHMWPE was present, regardless of processing condition or the use of irradiated or nonirradiated materials. The values of yield strength (Figure 10b) showed similar aspects because the UHMWPE component restricted the material flow. For the unirradiated samples, the values of deformation of failure in the cases of P1, P2, and P3 (Figure 10c) were much higher with respect to the other two compositions, which may be ascribed to material strength values near their flow limits. An opposite behavior may be noticed for Young’s modulus (Figure 10d), when the interactions between components had a similar significance to that reported for deformation at failure.

The γ-irradiation produced the fragmentation of macromolecules, followed by two contradictory processes: the oxidation and recombination of free radicals and the reassembling of molecules happening simultaneously with crosslinking. The HAp loading as an inorganic filler was involved in the modification of the rate of oxygen diffusion and in the stabilization activity as well [2]. Rosemary extract acted efficiently on the oxidation delay. Consequently, their attendance modified the mechanical properties of the studied polymer blends. The values of tensile strength (Figure 10a) increased with the yield strength. The same consequences of crosslinking may be noticed for the other properties, which were modified in relation with the radiation-processing effects. These results have been previously reported for UHMWPE [74,75] and LDPE [76,77].

The exposed results are reliable proof for the implementation of radiation processing in the production of high-quality polyethylene-based products, which are appropriate materials for a large variety of items subject to the action of destructive agents working under hazardous energetic conditions.

### 3.5. Contact Angle

The influence of liquid surroundings on polymer surfaces makes a difference in the adhesion, penetration, and lifetime of products. The current behavior of any polymeric material is undoubtedly related to the humidity characterizing the environmental conditions [78]. Wettability controls the penetration of liquids into materials and determines material durability. Consequently, the evolution of product state is influenced by the intimate interaction between liquid superficial pellicles and the inner polymer layers that can be disturbed by this tunneling penetration [79,80].

**Figure 11:**
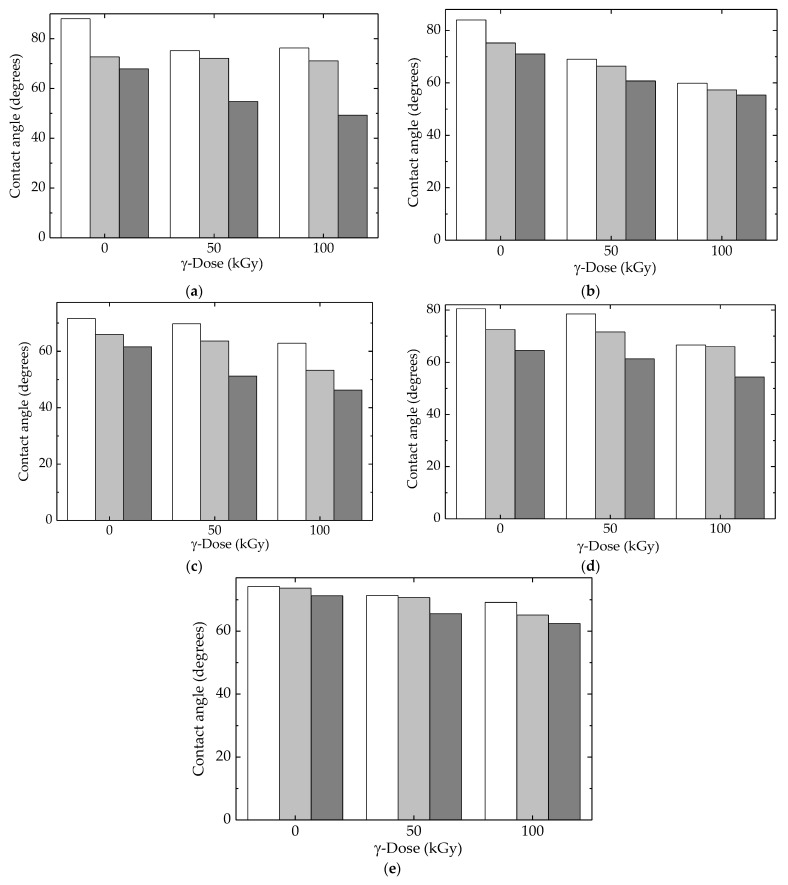
Contact angles calculated for all the investigated samples with (white) water, (light grey) glycerol, and (dark gray) ethylene glycol. (**a**) LDPE/UHMWPE, (**b**) LDPE/HAp, (**c**) LDPE/HAp/RM, (**d**) LDPE/UHMWPE/HAp, (**e**) LDPE/UHMWPE/HAp/RM.

**Figure 12:**
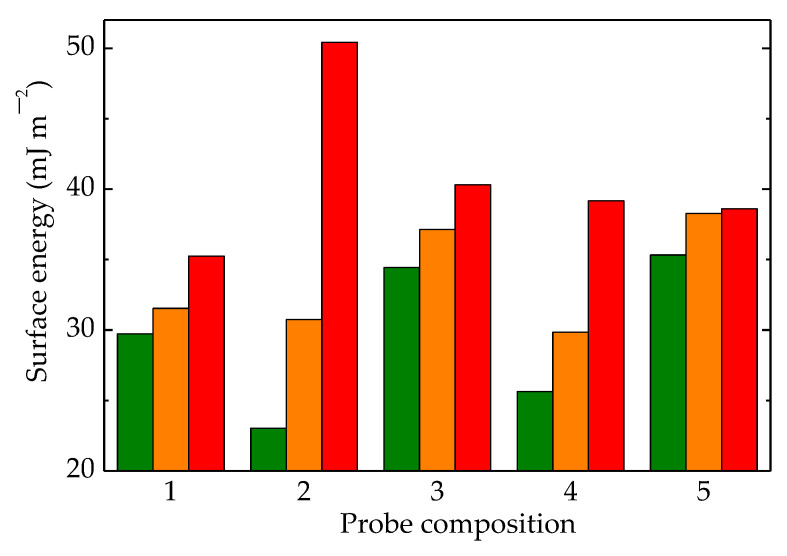
Surface energies calculated for LDPE/UHMWPE samples irradiated at (olive) 0 kGy, (orange) 50 kGy, and (red) 100 kGy. (1) LDPE/UHMWPE, (2) LDPE/HAp, (3) LDPE/HAp/RM, (4) LDPE/UHMWPE/HAp, (5) LDPE/UHMWPE/HAp/RM.

The contact angle measurements revealed the influence of molecule fragmentation, which produced an increase in wettability proved by the reduction in the contact angle values [81]. All the studied compositions of LDPE-based blends showed this tendency (Figure 11), but the diminishing degrees depended on the presence of the fillers, HAp and RM, which mitigated alteration in superficial tension. Figure 12 correlates the consequences of polymer degradation with superficial interaction when the droplets changed their adhesion angles in relation to the degree of scission. The peculiar actions of HAp and RM were revealed by their involvement in the degradation process when the inhibition of oxidation, as well as changes in the diffusion migration of oxygen and free radicals, remodeled the wettability behavior.

The interference of additional compounds was highlighted by the moderation of differences that existed between same-structured materials exposed at various γ-doses. The overall results related to the competition between the progress of oxidative degradation and the protection efficiency of additives was integrated into the evolution of the superficial behavior of the currently processed blends.

In Figure 12, the most striking differences in the values of surface energies were found for the LDPE/HAp formulation. This material had neither high-crystalline UHMWPE nor the efficient protector of RM. This demonstrates that the presence of these compounds in the structures of other formulations generated interaction effects with contacting liquids.

The liquids used were water, glycerol, and ethylene glycol with different surface tension values (72.8, 63.4, and 47.7 nN m^−1^, respectively) [82], and they could describe the responses of materials related to aging evolution when these materials operated under unlikely conditions. The smallest differences between the values of surface tension could be noticed for the complete composition, where UHMWPE, Hap, and RM were included in the LDPE matrices. Thus, the hydrophilicity was modified according to the contributions of all the particle components existing in the outer limit surface of the material. A certain composition led to a particular roughness [83], whose effects accompanied the contributions of the differences due to component alternation.

### 3.6. Biodegradability Testing

The biodegradability of polymeric materials by means of microorganisms can take place through various mechanisms, such as enzymatic depolymerization or polymer degradation by enzymatic hydrolysis [84]. The role of these mechanisms is to obtain short-chain compounds or small molecules (oligomers, dimers, or monomers), easily assimilated by cells, as well as the induction of functional groups in the polymer structure that reduce the degree of hydrophobicity of a material [85,86].

In general, polyethylene-based materials are long-chain polymers with high molecular weights and high degrees of hydrophobicity, making them not susceptible to degradation by microorganisms [86,87]. However, the application of various physical or chemical treatments can induce changes in the degree of crystallinity, molecular weight, mechanical properties, or surface properties, which are strictly connected to increased rates of biodegradability [88].

**Figure 13:**
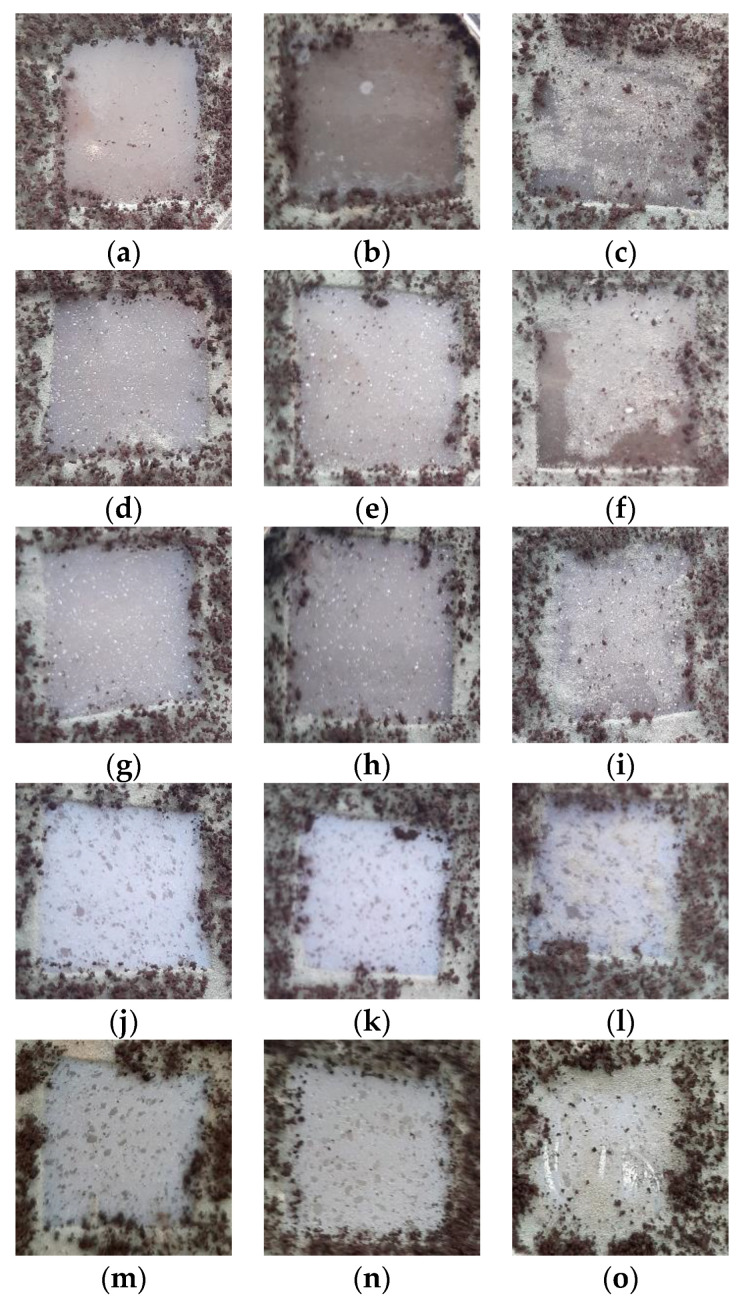
Images of the samples exposed to a fungi mixture after an incubation time of 28 days. Types of samples: (**a**–**c**) LDPE/UHMWPE, (**d**–**f**) LDPE/HAp, (**g**–**i**) LDPE/HAp/RM, (**j**–**l**) LDPE/UHMWPE/HAp, (**m**–**o**) LDPE/UHMWPE/HAp/RM. Irradiation doses: (**a**,**d**,**g**,**j**,**m**) 0 kGy, (**b**,**e**,**h**,**k**,**n**) 50 kGy, (**c**,**f**,**i**,**l**,**o**) 100 kGy.

In Figure 13, the images obtained of samples exposed to a fungi mixture after an incubation time of 28 days are presented. As can be observed, there were different fungi-covering degrees as a function of both sample compositions and irradiation dose. Thus, the greatest biodegradation susceptibility was observed for the LDPE/UHMWPE and LDPE/UHMWPE/HAp blends irradiated at 100 kGy, with more than 80% of the surface attacked by fungi. In similar conditions, LDPE/UHMWPE/HAp/RM presented about 50% and, for LDPE/HAp and LDPE/HAp/RM, about 25% of their surfaces were covered by fungi. It must be noted that, after 7 days, only the edges of sample were attacked by fungi, with the most significant differences between samples observed starting at the 14th day.

The consequences of γ-irradiation as an accelerated degradation process were found through the availabilities of the materials to bioattack. The visual inspection placed the biodegradability of the studied samples in the following order:

LDPE/UHMWPE/HAp > LDPE/UHMWPE > LDPE/UHMWPE/HAp/RM > LDPE/HAp > LDPE/HAp/RM

The increase in the degree of biodegradability with irradiation dose was a consequence of radiation-induced degradation (probably chain scissions) [89,90], which created favorable conditions for the development of fungi. The resulting compounds with low molecular weights were easily assimilated by cells, and the induction of functional groups with oxygen (i.e., carbonyl and carboxyl, as shown in the FTIR study) increased the surface hydrophilicity of the materials and improved the ability of the fungal strains to form a biofilm on polyethylene [90,91].

The detailed analysis of the biodegradation of polymer composites is an up-to-date subject that reveals the necessity of this kind of study to demonstrate the durability of materials [92]. The evolution control of biodegradability is a main purpose in applications of polymers, especially in the medical field [93].

### 3.7. SEM

**Figure 14:**
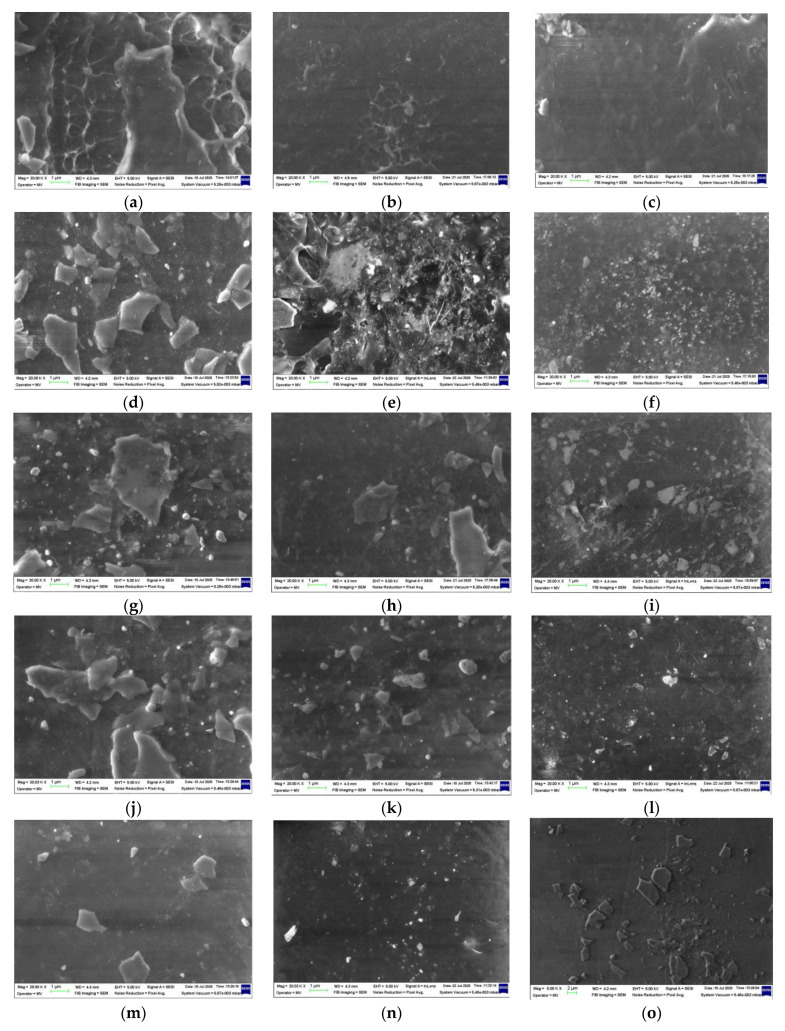
SEM images of the radiation-processed LDPE-based blends. Types of samples: (**a**–**c**) LDPE/UHMWPE, (**d**–**f**) LDPE/HAp, (**g**–**i**) LDPE/HAp/RM, (**j**–**l**) LDPE/UHMWPE/HAp, (**m**–**o**) LDPE/UHMWPE/HAp/RM. Irradiation doses: (**a**,**d**,**g**,**j**,**m**) 0 kGy, (**b**,**e**,**h**,**k**,**n**) 50 kGy, (**c**,**f**,**i**,**l**,**o**) 100 kGy. Magnification: 20 k.

The SEM images obtained by the morphological investigation are presented in Figure 14. Some peculiarities related to the modifications occurring in the mass of the polymer materials could be analyzed as the results of the radiolysis process [94]:

- The free radicals resulting from molecular scissions were the intermediates that were involved in several decay reactions according to the degradation mechanism [56];
- The material densification illustrated by the blackened images was the consequence of crosslinking. When the rosemary extract was present, the process took place more evidently due to the stabilization activity of polyphenol components;
- Decrease in the size of HAp particles was also a noticeable effect, which could be ascribed to the interactions between them and radicals that penetrated the surface through the material holes;
- Energy transfer occurred in the irradiated materials, stimulating the structural strengthening [95], which improved material durability. This behavior was also sustained by chemiluminescence measurements that provided direct information on the intimate changes in polymer configurations;
- The SEM images (Figure 14) are relevant proof for the involvement of the added compounds in the evolution of the material aging that took place at various rates when the polymer products were subjected to stressing actions.

## 4. Conclusions

The current study presents useful information regarding the radiation processing of various LDPE blends, where UHMWPE, hydroxyapatite, and rosemary extract completed the formulations. This technological procedure proved its benefits when the two polyethylene materials were protected by the named additives. The irradiation technique demonstrated their availability for the production of resistant materials. The relevant oxidation stability maintained even at high-irradiation γ-doses recommends these formulations as appropriate compositions for the manufacture of a large variety of items, including pipes and sealing O-rings or stoppers; protective sheets and common commodities; cases and bottles for food handling and beverages; and construction items and elastic buffers. Based on stabilization with rosemary extract, these compositions, especially LDPE/UHMWPE/HAp/RM, may be processed for the production of a large spectrum of items that are permanently in contact with people. The ecological features of these compositions are essential for applications in polymer recycling, when the valorization of polymer wastes becomes a part of human attitudes with respect to nature. The present results indicated that these kinds of materials may improve their lifespans through γ-processing. The activation energy values required for oxidative degradation were appropriate for the characterization of pertinent stability over long periods of operation. The contributions of the additives to the extension of stability consisted of their intimate interactions with free radicals, which were hindered to further promote material oxidative degradation. The proposed solutions may be taken into consideration for the manufacture of a large variety of products with foreseen durability.

## Figures and Tables

**Table 1:** Activation energies required for the radiation oxidation of LDPE/UHMWPE compositions irradiated at 50 kGy.

Sample	OIT (min)				Correlation Factor	E_a_ (kJ mol^−1^)
	160 °C	170 °C	180 °C	190 °C		
LDPE/UHMWPE	462	258	104	-	0.99410	121
LDPE/HAp	415	198	99	-	0.99998	116
LDPE/HAp/RM	-	480	186	109	0.98922	127
LDPE/UHMWPE/HAp	-	385	172	81	0.99998	132
LDPE/UHMWPE/HAp/RM	-	634	288	124	0.99952	139

## Data Availability

The data presented in this study are available on request from the corresponding authors.
